# The relationship between organized violence, family violence and mental health: findings from a community-based survey in Muhanga, Southern Rwanda

**DOI:** 10.3402/ejpt.v4i0.21329

**Published:** 2013-11-13

**Authors:** Heide Rieder, Thomas Elbert

**Affiliations:** Department of Psychology, University of Konstanz, Konstanz, Germany

**Keywords:** Child maltreatment, psychopathology, genocide, descendants, Rwanda, intergenerational, cycle of violence

## Abstract

**Background:**

The relationship between organized violence and family violence, and their cumulative effect on mental health in post-conflict regions remains poorly understood.

**Objective:**

The aim of the present study was to establish prevalence rates and predictors of family violence in post-conflict Rwanda. And to examine whether higher levels of war-related violence and its socio-economic consequences would result in higher levels of violence within families and whether this would be related to an increase of psychological distress in descendants.

**Method:**

One hundred and eighty-eight parent–child pairs from four sectors of the district Muhanga, Southern Province of Rwanda, were randomly selected for participation in the study. Trained local psychologists administered structured diagnostic interviews. A posttraumatic stress disorder (PTSD) diagnosis was established using the PTSD Symptom Scale Interview (PSS-I) and child maltreatment was assessed by means of the Childhood Trauma Questionnaire (CTQ). Additionally, the Hopkins Symptom Checklist (HSCL-25) assessed symptoms of depression and anxiety in descendants.

**Results:**

Prevalence rates of child abuse and neglect among descendants were below 10%. Ordinal regression analyses revealed that the level of child maltreatment in descendants was predicted by female sex, poverty, loss of the mother, exposure to war and genocide as well as parents’ level of PTSD and reported child maltreatment. Poor physical health, exposure to war and genocide, parental PTSD symptoms, and reported childhood trauma were significantly associated with depressive and anxious symptoms, while only exposure to war and genocide and poor physical health predicted the level of PTSD.

**Conclusion:**

The results indicate that cumulative stress such as exposure to organized violence and family violence in Rwandan descendants poses a risk factor for the development of depressive and anxious symptoms. Besides the support for families to cope with stress, awareness-raising initiatives challenging the current discourse of discipline toward children in schools or at home need to be fostered.

Substantial evidence suggests an elevated risk for lasting mental health problems in those exposed to war and conflict. Children and adolescents are a particular vulnerable group and may not only be affected by direct exposure to organized violence but also by secondary adverse effects, such as familial economic decline, malnutrition, lack of education or lasting familial conflict (Joshi & O'Donnell, 2003; Shaw, 2000). Family stressors such as the loss of beloved ones, the absence of, for example, fathers or ruptures in daily routines may disturb their social–emotional and cognitive development at a similar magnitude and partly mediate the relationship between war exposure and mental health (Miller & Rasmussen, 2010).

In a context where the majority of a family is affected by organized violence, children may additionally be exposed to an increase of physical violence within the family. Parents’ psychological impairment and war-related abuse of alcohol (Catani, Jacob, Schauer, Kohila, & Neuner, 2008) often have an impact on parenting skills and potentially produce violent behavior inflicted on children. Haj-Yahia and Abdo-Kaloti (2003) reported high rates of witnessed inter-parental and parent-to-sibling aggression and of experienced aggression by parents and siblings from a sample of Palestinian secondary school children. The authors found rates of physical violence to be correlated with the number of political stressors a family was exposed to (e.g., arrest of family members or curfew) as well as with specific family stressors (low income and a large family size) and parental aspects (low level of education and a poor psychological adjustment). Following a review by Guruge, Tiwari, and Lucea (2011) on international perspectives of family violence, being half-orphaned, limited social support, gender inequalities, economic deprivation, and living in a rural area present additional risk factors for an increase of intrafamilial violence. Boys compared to girls thereby seem to be at higher risk to experience child maltreatment (Alyahri & Goodman, 2008; Catani et al., 2009).

To our knowledge, no attempt has been made so far to investigate the described relationship between exposure to organized violence and family violence and subsequent mental health problems in post-conflict Rwanda. The East African country Rwanda has suffered from several waves of extreme “ethnic” violence since its independence that culminated in genocide in April 1994 leaving nearly a million people dead. Displacement, taking refuge, economic decline, and community conflicts due to widowhood and large-scale incarcerations especially in the South, hosting an elevated number of people accused of having taken part in genocide, characterized its aftermath. The psychological and somatic sequelae of these events are reflected in high rates of pathology in adults (Munyandamutsa, Nkubamugisha, Gex-Fabry, & Eytan, 2012; Pham, Weinstein, & Longman, 2004) as well as children and adolescents who survived the slaughters (Bolton, Neugebauer, & Ndogoni, 2002; Dyregrov, Gupta, Gjestad, & Mukanoheli, 2000; Schaal & Elbert 2006). According to Schaal, Duzingizemungu, Jacob, and Elbert (2011), Rwandan genocide widows and orphans represent particular vulnerable groups. The authors demonstrated that besides exposure to traumatic events, the major part of the level of genocide-related posttraumatic stress disorder (PTSD) was explained by a poor physical health status in both widows and orphans. Physical illness thereby can be understood as either a predictor or a consequence of PTSD (Engelhard, Van den Hout, Weerts, Hox, & Van Doornen, 2009). Ongoing chronic pain as reported by many genocide survivors seems to maintain PTSD symptomatology over a long period of time (Sharp & Harvey, 2001). Fourteen years after the genocide, Munyandamutsa et al. (2012) demonstrated that those with PTSD still showed lower scores of physical health on all dimensions compared to those without PTSD. In a poor country such as Rwanda, socio-economic hardship and mental health problems might additionally be associated (Lund et al., 2010). However, data from Rwanda are inconsistent with regard to this relationship and suggest that poverty rather functions as a mediator between physical health and mental health outcomes (Rieder & Elbert, 2013; Schaal et al., 2011). Recently, Betancourt et al. (2012) described the deterioration of the social cohesion, poverty, and the caregiver's illness or death as special threats to Rwandan children's health and wellbeing.

As mentioned above, Rwanda has been repeatedly affected by violent episodes for the last five decades. In particular, elderly Rwandans have not only lived through genocide but have also been exposed to various situations of conflict and war that may have affected their mental health thereby increasing the risk of perpetrating violence against family members or transmitting traumatic experiences to the next generation. Gupta, Reed, Kelly, Stein, and Williams (2010) demonstrated the impact of exposure to human rights violations of South African men on their violent behavior against their female partners. In a recent study from Gaza on intergenerational effects of parental war trauma on offspring's mental health, Palosaari, Punamäki, Qouta, and Diab (2013) showed that the father's past war experiences correlated positively with the child's level of depression and PTSD while being mediated by psychological maltreatment. An increased level of family violence as a result of a family member's exposure to organized violence could be explained by the following two hypotheses: on the one hand experiencing organized violence may raise the vulnerability of a parent to perpetrate violence against a family member while this relationship moreover may be mediated by posttraumatic stress symptoms (Shaw, 2000). A disturbed affect regulation in the traumatized parent may result in inappropriate rearing practices or violent behavior inflicted on children as described by various US studies, for example, on combat-related PTSD (Dekel & Monson, 2010; Jordan et al., 1992). On the other hand, many traumatized children present a higher level of behavioral disturbances, emotional problems, or attention deficits that in turn provoke aggressive and punishing behavior in a parent. In Rwanda, this question has been discussed with particular regard to gender differences, as a majority of genocide survivors are female whereas a majority who participated in genocide are male. Recent research has shown that the latter still display a significant level of PTSD after being released (Rieder & Elbert, 2013) and reintegration into former families may cause familial conflicts. Women left alone, whether due to being widowed (Brounéus, 2008) or due to having their husbands in prison and lacking social support may conversely be unable to answer their children's need, thereby increasing the risk of neglect and abuse especially if they have to take care of other family members or orphans.

In addition, there is evidence suggesting that parents’ current abusive behavior may be triggered by their own early adverse experiences as suggested by the “cycle of violence” hypothesis (Widom, 1989). It assumes that a predisposed abusive parent transfers its own experiences to the next generation turning oneself from a victim position as a child to a perpetrator as an adult. Previous research on intimate partner violence has critically discussed the potential impact of child abuse on later violence perpetration inside a couple or family (Heyman & Smith Slep, 2002).

However, studies examining the relationship between Rwanda's history of organized violence and family violence are missing and there is still little epidemiological information of the magnitude of the latter in general. A nationwide Rwandan study of more than 1000 adults between 20 and 64 years reported 47% of physical abuse (beating and assaulting), five times more often perpetrated by men compared to women, 21.6% of child sexual abuse and 14% of rape in young women and girls (NURC, 2008). In a recent study on gender-based violence, the highest rate was found in the urban district Kicukiro, part of Kigali Province even while the authors argue that differences between rural and urban areas were rather small (Rwandan's Men's Resource Centre, RWAMREC, 2010). In line with previous research, the authors explained that boys compared to girls experienced more family violence due to differences in the way education and discipline principles are exerted on children.

The first aim of the present study was to assess prevalence rates and risk factors of family violence in Rwandan families, exploring a representative sample of Muhanga district, Southern Province, as an example, hosting both genocide survivors and genocide suspects. The second aim was to assess the level of depressive, anxious and PTSD symptoms and to examine variables associated with mental health outcomes among descendants. By investigating the interplay of organized violence and family violence and subsequent mental health problems, we aimed to highlight the complexity of stress that a child growing up in a post-conflict society can be confronted with. We hypothesized that male sex, poverty, being half-orphaned, living in an urban area, a high number of siblings, and previous exposure to organized violence would lead to higher levels of reported child maltreatment. We further expected that the parents’ prior victimization during childhood and their current level of PTSD symptoms would contribute to explain its variance. Second, we assumed that poverty, the status of being half-orphaned, poor physical health, and having experienced cumulative exposure to family violence and organized violence would result in higher levels of depressive, anxious, and PTSD symptoms.

## Method

### Sampling and procedure

Eligible participants were genocide survivors and former prisoners accused of participation in genocide, and their children either born before or after the genocide. Genocide survivors were defined as individuals who were targeted throughout the genocide because of their Tutsi “ethnicity,” in Rwanda called *rescapé*. Former prisoners, the *génocidaires*, were defined as released prisoners who, during the past 16 years, were incarcerated for and accused of genocide-related crimes. The present publication focuses on the level of reported childhood trauma and the mental health situation of descendants while hereby neglecting the family's background of victimhood or participation in genocidal violence.

In total, 188 parent–child pairs were included. Inclusion criteria for the parent generation were that they had resided in Rwanda in 1994 and that they were at least 18 years old at that time. The parent sample was between 30 and 81 years old and consisted of 72 women (41.9%) and 100 men (58.1%). Descendants were child and adolescent survivors of genocide (19–31 years old) and children born after 1994 had to be between 13 and 15 years. One hundred descendants were female (53.2%) and 88 were male (46.8%). There was no significant difference in gender between descendants born before versus after 1994, but descendants born before 1994 were more often half-orphaned (*χ* ^*2*^(1,188) = 15.9, *p*<0.001), and had more years of schooling (*U*=2550.5, *p*<0.001). Further demographic characteristics for all generations can be drawn from Table 1.

**Table 1:** Socio-demographic characteristics, mental health, and childhood trauma in Rwandans

	Parent generation (*n*=172)	Descendants (*n*= 188)
Age M (SD, range)	52.7 (9.3, 30–81)	21.3 (5.9, 13–31)
Sex% (*n*)		
Women	41.9 (72)	53.2 (100)
Men	58.1 (100)	46.8 (88)
Half-orphan % (*n*)	n.a.	37.8 (71)
Mother alive	n.a.	94.1 (177)
Father alive	n.a.	68.1(128)
Number of siblings M (SD, range)	n.a.	5.7 (2.2, 0–13)
Location of growing up after 1994*%* (*n*)		
One's own family	n.a.	90.3 (168)
Mother's family	n.a.	5.9 (11)
Father's family	n.a.	3.8 (7)
Current location (sector)*%* (*n*)		
Nyamabuye	31.4 (54)	30.3 (57)
Shyogwe	19.8 (34)	19.1 (36)
Cyeza	19.8 (34)	20.2 (38)
Muhanga	29.1 (50)	30.3 (57)
Years of schooling M (SD, range)	4.5 (3.5,0–14)	6.09 (3.2, 0–17)
Health problems*%* (*n*)		
Chronic pain	32.1 (55)	3.2 (6)
Chronic diarrhea	5.2 (9)	5.3 (10)
Disabilities	5.8 (10)	3.2 (6)
HIV	7.0 (12)	.5 (1)
Other illnesses	51.2 (88)	44.4 (83)
Exposure to war and genocide M (SD, range)	11.0 (3.8, 2–24)	6.0 (5.0, 0–18)
Losses in the family due to genocide M (SD, range)	8.4 (12.3, 0–70)	n.a.
PTSD		
Diagnosis*%* (*n*)	23.4 (40)	8.5 (16)
Severity score M (SD, range)	9.2 (11.0, 0–45)	3.4 (6.8, 0–40)
Anxiety		
Severity score M (SD, range)	16.3 (7.7, 10–40)	13.1 (5.2, 10–32)
Depression		
Severity score M (SD, range)	19.9 (7.2, 15–55)	16.6 (3.6, 15–38)
Childhood trauma M (SD, range) (%)	32.6 (8.6, 25–74)	33.3 (7.7, 25–103)
Physical abuse	5.7 (1.9, 5–18), 3.5	5.5 (1.7, 5–24), 2.1
Emotional abuse	6.0 (2.9, 5–18), 4.1	5.8 (2.1, 5–24), 1.1
Physical neglect	7.1 (2.4, 5–18), 9.3	6.9 (2.0, 5–15), 9.6
Emotional neglect	8.9 (3.1, 5–23), 6.4	9.4 (2.3, 5–17), 3.1
Sexual abuse	5.3 (1.0, 5–12), 4.1	5.5 (2.1, 5–25), 6.4
Minimization/denial M (SD, range)	1.1 (0.98, 0–3)	0.7 (0.7, 0–3)
MD ≥ 1 in% (*n*)	69.9 (119)	59.4 (110)

n.a.=not assessed.

The present study was conducted from May to July 2010 in the Southern province of Rwanda, Muhanga district, located 45 km from the capital Kigali. The survey was approved by the National Institute of Statistics of Rwanda (NISR) and by the Ethical Review Board of the University of Konstanz, Germany. Local authorities of the selected district and sectors provided research permits in the local language Kinyarwanda to facilitate access to the interviewees. Interviews were conducted individually in the respondents’ home by seven local BA-level psychologists from the National University of Butare, Rwanda who had participated in epidemiological surveys before and, from there, had received extensive training and experience in conducting structured clinical interviews. A clinical psychologist throughout the whole research period supervised interviewers. The survey was conceived as a community-based study and interviews were conducted in four randomly selected sectors applying a simple random sampling approach through numbering all sectors and selecting four without replacing them after their selection. In this way, three rural sectors (Cyeza, Shyogwe, and Muhanga) and one urban sector (Nyamabuye), representing the administrative center of Muhanga as part of the provincial town Gitarama were included. In all sectors, two quarters were randomly chosen and interviewers went door-to-door, starting at a convenient location within an assigned sector and approaching each subsequent house. The first adult who met inclusion criteria and had children within the required age ranges was interviewed. If more than one adult householder met inclusion criteria, one was randomly selected out of the two. If eligible parents had more than one child that fulfilled the criteria, one was chosen for participation and if parents had children that met criteria for both age ranges (either being born before or after 1994) both children were interviewed. Interviews were continued until the required number was attained. After having extensively informing the participants and having received written informed consent, interviews took one to two hours. At the end of the interview, participants received 1000 Rwandan Francs (about 1.30 Euro) as compensation for the time spent on the interview.

### Clinical assessments

All participants in the present survey were screened for PTSD and childhood trauma. Additionally, symptoms of anxiety and depression were assessed in all descendants.

Socio-demographic data included age, sex, half-orphan status, years of schooling, current location and location after the genocide, and physical health problems. An economic status was established consisting of the following variables: possessions (house, agriculture), any monthly monetary income, the capacity to satisfy the family's needs and facts on typical nutrition (number of meals, with or without proteins). Its index was built dividing their added *z*-transformed variables by the square root of their number. Physical health was assessed integrating six questions on common symptoms and syndromes in Rwanda (chronic pain, chronic diarrhea, tuberculosis, HIV, disabilities due to war violence or any other illness in the past 6 months) and compiling answers to an index of physical illness ranging from 0 to 6.

Exposure to trauma was assessed by means of an Event-Scale modified for the Rwandan context (Schaal & Elbert, 2006) containing 25 potentially traumatic stressors. The trauma load was estimated for all participants. The PTSD symptom severity was measured by use of the PTSD Symptom Scale—Interview (PSS-I, Foa & Tolin, 2000). Besides an overall severity score (ratings on a four-point Likert-type scale, possible sum score range from 0–51), a PTSD diagnosis according to the *DSM-IV* criteria was established. The Kinyarwanda version of the PSS-I was produced in a Ugandan refugee camp settlement close to the Rwandan border and showed satisfactory psychometric properties (Onyut et al., 2004).

Symptoms of depression and anxiety were assessed by means of the Hopkins Symptom Checklist (HSCL-25, Derogatis, Lipman, Rickels, Uhlenhuth, & Covi, 1974), one of the most frequently used instruments in transcultural research including the East African context. Severity scores (possible range for anxiety symptoms from 10 to 40 and for depressive symptoms from 15 to 60) were used for further analyses rather than relying on cut-off criteria (Ertl, Pfeiffer, Saile, Schauer, & Elbert, 2010; Rieder & Elbert, 2013).

The Childhood Trauma Questionnaire (CTQ, Bernstein & Fink, 1998), a self-report instrument, was used in its short form to assess traumatic experiences of abuse and neglect during childhood. It contains 25 statements to be rated on a five-point Likert-type scale with regard to the frequency with which it appeared during childhood. Its five different dimensions of childhood trauma are as follows: physical and emotional abuse, physical and emotional neglect, and sexual abuse. A three-item minimization or denial scale was included as a validity scale that indicates a potential underreporting of maltreatment (false negatives) or idealizing of the family of origin. Scores of responses were dichotomized and summed up to a scale sum score. A total of one suggests possible underreporting of maltreatment and a score of three indicates extreme denial. For the present study, a Kinyarwanda version of the questionnaire was produced using independent translation and blind reverse translation based on the validated French version of the CTQ, which showed very good psychometric properties (Paquette, Laporte, Bigras, & Zoccolillo, 2004). Both versions were later on examined for discrepancies and extensively discussed before its final approval. In the present study, raw scale scores and prevalence rates were established. With regard to sub-Sahara Africa, data based on the CTQ are reported from clinical and non-clinical samples from Togo and South Africa (Kounou et al., 2012; Suliman et al., 2009). To establish CTQ prevalence rates, a procedure suggested by Kounou et al. was followed: abuse was classified if the score was placed in the moderate or severe level, for example, cut-off scores were 13 or higher for emotional abuse, 10 or higher for physical abuse, 8 or higher for sexual abuse, 15 or higher for emotional neglect, and 10 or higher for physical neglect. Subscales showed low (Cronbach's *α*=0.56 for emotional neglect and 0.61 for physical neglect) to very good (0.83 for physical abuse and 0.90 for sexual abuse) internal consistency.

### Statistical analyses

First, levels of reported child maltreatment and psychopathology (PTSD, depression, anxiety) were described using frequencies, mean scores, and standard deviations. Second, Spearman's rank correlations were calculated to further analyze risk factors with regard to the overall level of child maltreatment. Third, three sets of regressions analyses were run in order to test independent predictors of the CTQ sum score, of the HSCL-25 sum score and of the PSS-I sum score. As the assumptions to run linear regression analyses were not fulfilled by the data (e.g., residuals were not normally distributed), ordinal regression analyses were calculated instead. The regression model on the CTQ sum score was calculated by entering the following variables: sex, economic status, status of being half-orphaned, number of siblings, location (urban vs. rural), exposure to war and genocide, parental exposure to child maltreatment and parental PTSD. Using the HSCL-25 sum score and the PSS-I sum score as outcome variables, the following variables were entered in the analyses: sex, physical health, being half-orphaned, economic status, exposure to war and genocide, reported childhood trauma and parental PTSD. Data analyses were performed using SPSS^®^ software, version 21.

## Results

### Trauma exposure

Respondents were exposed to a wide range of traumatic stressors. The parent generation recalled an average of 11 different types of traumatic events in a lifetime (SD = 3.8, range 2–24). 19.3% reported witnessing the killing of someone, 15.3% physical attack and 11.4% seeing dead and mutilated bodies to be the worst and most stressful experiences. Descendants born before 1994 experienced about 8.3 traumatic events (SD = 4.2, range 0–18). Again, witnessing the killing of someone (18.6%), seeing dead and mutilated bodies (10.9%), and being attacked with a weapon (10.9%) were among the most stressful events. Descendants born after 1994 experienced an average of 0.8 traumatic event types (SD = 1.3, range 0–7) and referred to witnessing and experiencing life-threatening accidents as the worst events.

### Level of distress

The prevalence rate for current PTSD in the parent generation was 23.4% (*n*=40) and 12.4% (*n =*16) in the group of descendants born before 1994. None of the descendants born after 1994 fulfilled the criteria for PTSD. Descendants born before 1994 showed an HSCL-25 mean score of 31.1 (SD = 9.1, range 25–62) and descendants born after 1994 showed a mean score of 26.6 (SD = 3.4, range 25–42). Both mean scores differed significantly (*U*=2590.0, *p*<0.001). Physical health problems among descendants were mainly related to common symptoms and syndromes, such as malaria, headaches, abdominal and back problems, and nervous crises. Symptom scores of all disorders assessed are shown in Table 1.

### Child maltreatment

Mean scores of reported childhood maltreatment were 32.6 (SD = 8.6, range 25–74) for the parent generation and 33.3 (SD = 7.7, range 25–103) for the entire sample of descendants, that is, 34.3 (SD = 8.7, range 25–103) for descendants born before 1994 and 30.8 (SD = 3.5, range 25–45) for descendants born after 1994. Descendants born before 1994 displayed a significantly higher level of maltreatment compared to those born after (*U*=2097.0, *p*<0.001). Rates of the minimization and denial scale showed that a great majority of both parents and descendants possibly underreported the level of experienced maltreatment. For scale scores and percentages of all CTQ dimensions, see Table 1.

### Risk factors and prediction of child maltreatment in descendants

When examining risk factors of child maltreatment, exposure to war and genocide (*ρ*=0.35, *p*<0.001), showed the strongest correlation followed by economic status (*ρ*= − 0.30, *p*<0.01), parental exposure to child maltreatment (*ρ*=0.21, *p*<0.001), and parental PTSD symptoms (*ρ*=0.21, *p*<0.01). An ordinal regression model analysis was run to identify independent contributions of risk factors to the level of childhood maltreatment. The final model explained 27% of the variance in the outcome variable. Significant predictors can be drawn from Table 2.

**Table 2:** Beta coefficients and correlations coefficients resulting from bivariate correlations and ordinal regression analysis on the amount of childhood trauma (CTQ sum score) reported by descendants (*N*=188)

	*β*	*ρ*
Sex (female)	0.84**	0.12
Mother alive (no)	1.39*	0.13
Father alive (no)	−0.09	0.16*
Number of siblings	0.08	0.04
Economic status	−0.08*	−0.30***
Location (urban)	−0.18	−0.11
Exposure to war/genocide (no of traumatic events)	0.13***	0.35***
Parents’ exposure to childhood maltreatment (CTQ sum score)	0.08***	0.27***
Parents’ PTSD (symptom severity)	0.03*	0.21**

* *Note:* *p*<0.05

** *p*<0.01

*** *p*<0.001.

Spearman's *ρ* resulting from bivariate correlations.

Full model's adjusted *R* ^2^=0.29; (9,188) = 62.9, *p*<0.0001.

Significant predictors are in bold.

### Prediction of psychopathology in descendants

As shown in Table 3, individual predictors of distress were analyzed by calculating two ordinal regression analyses on the HSCL-25 sum score and the PSS-I sum score. The first model explained 39% of the variance. Descendants with poor physical health, who reported a great exposure to war and violence, a high level of child maltreatment and whose parents currently suffered from PTSD were more likely to show an increased level of depressive and anxiety symptoms. The second model explained 54% of the variance in the PSS-I sum score of those participants who had experienced at least one traumatic event. Exposure to war and genocide and physical health were significant predictors.

**Table 3:** Beta coefficients and correlations coefficients of psychopathology in descendants resulting from ordinal regression analyses using the HSCL-25 sum score for all descendants and using the PSS-I sum score for descendants who experienced at least one traumatic event type as outcome variables

	HSCL-25 (*n*=188)^1^		PSS-I (*n*=158)^2^	
	
Predictors	*β*	*ρ*	*β*	*ρ*
Sex (female)	0.08	0.07	0.38	0.04
Poor physical health	0.75**	0.35***	0.92***	0.38***
Half-orphan (yes)	0.32	0.33***	0.37	0.40***
Economic status	0.03	−0.17*	−0.04	−0.22**
Exposure to war/genocide (no of traumatic events)	0.16**	0.48***	0.36**	0.71***
Exposure to childhood maltreatment (CTQ sum score)	0.08**	0.38***	0.01	0.26***
Parents’ PTSD (symptom severity)	0.03*	0.29***	0.003	0.29***

* *Note:* *p*<0.05

** *p*<0.01

*** *p*<0.001.

CTQ = Childhood Trauma Questionnaire.

1 Model (HSCL-25 sum score): Full model's adjusted *R* ^2^=0.39; χ^2^ (7, 188) = 92.2, *p*<0.0001.

2 Model (PSS-I sum score): Full model's adjusted *R* ^2^=0.54; χ^2^ (7, 158) = 118.7, *p*<0.0001.

Significant predictors are in bold.

## Discussion

The present study examined the relationship between exposure to organized violence, family violence, and related mental health outcomes, such as PTSD, depression, and anxiety in a randomly selected sample of Rwandan descendants in Muhanga district, 16 years after the genocide. Female sex, poverty, the loss of one's mother, and cumulative stress consisting of exposure to organized violence, parents’ level of PTSD and parent's level of exposure to childhood trauma predicted the amount of reported child maltreatment in descendants. Subsequently, depressive and anxious symptoms were associated with poor physical health, exposure to diverse forms of violence and again parent's level of PTSD. PTSD showed only to be related with exposure to organized violence and poor physical health.

Rwandan child and adolescent survivors of genocide had been exposed to about eight types of traumatic stressors and showed a PTSD rate of 12.4%. Several studies have already demonstrated the massive trauma confrontation in Rwandan youth and its consequences on mental health (Dyregrov et al., 2000; Neugebauer et al., 2009; Schaal & Elbert, 2006). The level of depressive and anxious symptoms among all descendants was comparable to data from Rwandan refugees in Uganda using the same instrument (Onyut et al., 2009). However, descendants born 1–3 years after the genocide showed no clinically significant level of PTSD and manifested lower symptom levels of depression and anxiety compared to those born before 1994. An obvious reason for the comparatively better health may be that the post-conflict generation experiences less, while the war generation experiences more (10 times more in the present samples) stress and adversity.

Rates for reported childhood trauma were unexpectedly low compared to earlier studies from post-conflict regions (e.g., Catani et al., 2008). A recently published nationwide study conducted by the Rwanda Men's Resource Centre (RWAMREC, 2010) that included 3612 adults aged between 18 and 60 years showed that more than 50% of the respondents recalled experiences of sexual and physical violence throughout their childhood, whereas in our sample rates for subscales were below 10%. However, the total CTQ mean score was comparable to a study from South African youth and to a clinical sample of adults in Togo (Kounou et al., 2012; Suliman et al., 2009). Different explanations for this discrepancy are possible: firstly, in the present study a child growing up without its mother showed to be more at risk to be exposed to abuse and neglect. Experiencing less protection and attention due to the loss of their mother may put children into a vulnerable position. Children possibly have to take over mother's tasks such as domestic work and cultivation of food crops and at the same time are at higher risk to be physically abused or emotionally neglected. This, in turn, could indicate that the elevated number of half-orphans in our sample accounts for the general low levels of child maltreatment as fathers are missing in 32% of the families and less perpetration of violence inside the families may therefore take place (RWAMREC, 2010). Secondly, the high percentage of denial of abuse found among both generations (two to three times higher compared to a sample from South Africa) suggests that older participants, in particular, may have chosen an idealized frame of reference when reporting on their childhood. Internally comparing the survival of extreme violence such as genocide to early adverse experiences may have yielded distorted information, as the latter may have appeared less disturbing and threatening. And thirdly, the general higher acceptance of interfamilial violence in Rwanda, while individually being rejected, might be reflected by the present data given that violence at home might not be perceived as such (RWAMREC, 2010).

Exposure to war and genocide showed to be a risk factor of child maltreatment suggesting that children experiencing organized violence may also be confronted with other forms of violence. The finding that the parents’ level of PTSD additionally contributed to explain the variance in family violence among descendants further supports this. As stated above, a great portion of the sample was half-orphaned mainly due to the death of their fathers. While generally lower levels of child maltreatment are assumed in those families, the matter of parental PTSD still needs to be considered as it may affect the parent–child relationship. This was previously explained by Margolin & Vickerman (2007) who stated that mothers with PTSD may be less emotionally available for their children and may be more impulsive in their actions toward them. Studies including both parents of a child are needed here in order to understand differences of the impact of parental psychopathology.

As assumed, poverty did account for an increased level of family violence. Rwanda's peasant population was already characterized by poverty and starvation before the genocide but its direct aftermath produced new challenges as a large portion of the population was widowed and female-headed households were found throughout the entire society. Women did not yet have the right to inherit land and eventually finished in an impoverished environment (Schindler, 2010). Traditionally, partner relationships in Rwanda are marked by power inequality between the genders but social realities after 1994 gave women more rights through new laws and policies. In contrast, men who formerly perceived themselves as the defender of their country and families, potentially lacked self-esteem due to their incapacity of providing safety in 1994. Therefore, women started gaining power and the “deeply ingrained” gender model transmitting, for example, violence against women as a regulating mechanism within the household, a fact accepted as such by a large portion of both women and men, subsequently was put into question (RWAMREC, 2010). Today, employed women living with unemployed men as well as women with higher incomes show to be endangered to experience intimate partner violence (Finnoff, 2010; RWAMREC, 2010). This illustrates the complexity and high interrelatedness of a family's economic situation, changing family structures, and family violence in post-conflict Rwanda. Children therefore continue to be at risk of witnessing inter-parental violence and experiencing domestic violence while specific underlying family mechanisms might have changed.

Other family characteristics such as a high number of children did not present an individual predictor of violence within the family when controlled for further socio-demographic characteristics. Possibly, growing up in the context of an extended family as common in Rwanda might function as a protective factor as children are not necessarily exposed to their parents only but may choose where to go to and get support from other significant adults. Contrary to our hypothesis and to findings from a previous Rwandan study (RWAMREC, 2010), female sex and not male sex presented a risk factor of experiencing child maltreatment. Further data collection is needed here to identify potential mediating factors, in particular with regard to the parent–child relationship.

Parents’ own experiences of childhood maltreatment individually contributed to explain the variance of reported family violence in descendants. This substantiates our assumption that prior victimization of parents in early childhood may increase the risk of violence perpetration within a family. These results are in line with an earlier cited Rwandan study (RWAMREC, 2010) demonstrating that men's risk of perpetrating violence against women was linked to their own experience of physical abuse and of witnessing inter-parental violence during childhood. Furthermore, their exposure to genocide, while this has not been assessed systematically, showed to be related to later violence perpetration compared to those who had not experienced genocide. However, Saile, Neuner, Ertl, and Catani (2013) who examined intimate partner violence in a sample of couples from Northern Uganda could not replicate those findings. The current data do not allow us to create a more complex model of intergenerational transmission of abuse especially as no behavioral data were assessed in descendants (Thornberry, Knight, & Lovegrove, 2012). Nonetheless, it shows some evidence for the “cycle of violence” hypothesis with regard to the overall level of reported child maltreatment.

As expected, the level of depressive and anxious symptoms was predicted by both reported child maltreatment and exposure to organized violence. This is consistent with previous research (Shaw, 2000; Turner, Finkelhor, & Ormrod, 2006). In contrast to that, the variance in PTSD symptoms was only explained by exposure to war and genocide but not by childhood trauma. Even while evidence is growing that exposure to interpersonal violence, either witnessed or experienced, may result in an increased level of PTSD in youth (Margolin & Vickerman, 2007), our data rather showed a strong dose–response relationship between organized violence and PTSD. Thus, the data illustrate the ongoing detrimental effect of organized violence on traumatized youth's health, 16 years after the genocide even when controlled for post-conflict adversities.

A poor physical health status was significantly linked to psychopathology in descendants. Physical illness was commonly found to be associated in particular with PTSD and could be confirmed as a risk factor in our sample (Munyandamutsa et al., 2012; Schaal et al., 2011; Schnurr & Jankowski, 1999). Economic hardship did not present as a predictor of mental health problems. Boris et al. (2008) who reported data from youth headed households in the Southwest of Rwanda also showed that a poor physical health and social marginalization but not economic aspects such as hunger were associated with depressive symptoms.

Inconsistent results were found with regard to the impact of the parent's level of suffering from PTSD on descendants’ mental health as it showed to be a predictor of the level of depression and anxiety symptoms but not PTSD. In part, this is in line with a study from war-affected families from Gaza where both parents and children were exposed to traumatic experiences (Thabet, Abu Tawahina, El Sarraj, & Vostanis, 2008). The authors found that war trauma and parents’ emotional responses in the form of PTSD and anxiety were significantly associated with children's anxiety symptoms and PTSD. Schick, Naser, Klagfofer, Schnyder, and Müller (2013) recently found that only paternal PTSD but not maternal PTSD was related to depressive symptoms in children when examined in a sample of 51 war-affected families 11 years after conflict in Kosovo. However, based on our data we assume that the relationship between parental PTSD and depressive symptoms in descendants might be mediated by parents’ way of dealing with trauma-specific losses. A “depressed or irritable family environment” as discussed by Shaw (2000) may severely affect children and in turn result in an increase of a child's psychopathology. Similar findings were reported by Smith, Perrin, Yule, and Rabe-Hesketh (2001) from a Bosnian sample. The authors found that the children's level of exposure explained the greatest part of the variance of children's level of PTSD while the mother's mental health explained depressive symptoms in children.

The current study presents with several limitations. Interviewing triples (father, mother, child) would enable us to further differentiate between mothers’ and fathers’ specific influence on descendants. Still, the inclusion of parents and children offers insight into the interdependence of genocidal violence and childhood trauma, although prevalence rates of the latter may have been underestimated due to denial. The use of the CTQ is critical and further research is needed here to clarify if its application is appropriate in specific with regard to the East African context. Findings are representative for central Gitarama and might not necessarily hold for the entire country. Also, we did not assess the primary caretaker of the descendants. Additional information of the family context is needed here to identify parental influences’ on their children as those do not necessarily grow up with their parents or spend more time with other members of the extended family.

## Conclusion

Our initial assumption that exposure to organized violence will enhance the level of family violence was confirmed. Findings further demonstrated that stressors such as poverty and parental psychopathology additionally may affect the family system. As a consequence, such a cumulative stress poses a risk factor for the future development of descendants who may be more likely to develop depressive and anxious symptoms. Results of the present study indicate that a wider focus including the family level and its socio-economic environment needs to be taken into account when dealing with consequences of war and conflict. In addition to psychotherapeutic treatment for traumatized individuals, families need support to cope with stress within the family system. Moreover, awareness-raising initiatives challenging the current discourse of discipline toward children in schools or at home and violence between partners need to be fostered.
